# Re-engaging with the past: recapitulation of encoding operations during episodic retrieval

**DOI:** 10.3389/fnhum.2014.00351

**Published:** 2014-05-27

**Authors:** Alexa M. Morcom

**Affiliations:** Centre for Cognitive Ageing and Cognitive Epidemiology, Department of Psychology, University of EdinburghEdinburgh, UK

**Keywords:** episodic memory, encoding, retrieval, reactivation, transfer-appropriate processing, subsequent memory, fMRI, reinstatement

## Abstract

Recollection of events is accompanied by selective reactivation of cortical regions which responded to specific sensory and cognitive dimensions of the original events. This reactivation is thought to reflect the reinstatement of stored memory representations and therefore to reflect memory content, but it may also reveal processes which support both encoding and retrieval. The present study used event-related functional magnetic resonance imaging to investigate whether regions selectively engaged in encoding face and scene context with studied words are also re-engaged when the context is later retrieved. As predicted, encoding face and scene context with visually presented words elicited activity in distinct, context-selective regions. Retrieval of face and scene context also re-engaged some of the regions which had shown successful encoding effects. However, this recapitulation of encoding activity did not show the same context selectivity observed at encoding. Successful retrieval of both face and scene context re-engaged regions which had been associated with encoding of the other type of context, as well as those associated with encoding the same type of context. This recapitulation may reflect retrieval attempts which are not context-selective, but use shared retrieval cues to re-engage encoding operations in service of recollection.

## INTRODUCTION

Episodic memory is thought to be a largely incidental record of daily life, and depends on the formation of associations between the different features of events and the context in which they occur (Tulving, 1983; Eichenbaum, 1999). Recollection of an event depends on the later reassembly of some subset of its unique constellation of features and context, given an appropriate cue (Tulving and Thomson, 1973; Morris et al., 1977). Neuropsychological and neurophysiological studies have established that forming and retrieving vivid memories depends critically on the integrity of the hippocampus (see Spiers et al., 2001). Functional imaging data converge with this view, providing separate measures of the processes operating during encoding and retrieval (Fletcher et al., 1997; Eichenbaum et al., 2007), which have brought into focus the cortical activity underpinning specific memory experiences (Wheeler et al., 2000). A growing literature now demonstrates that recollection involves contextual reinstatement, associated with reactivation of the brain regions which were engaged during the original event. This is revealed in the selective reactivation at retrieval of neural activity associated with different dimensions of events: their sensory, emotional and cognitive contexts (for review see Rugg et al., 2008; Danker and Anderson, 2010). It is proposed that this neural reactivation supports recollection through the reinstatement of representations of event’s original features (Wheeler et al., 2000), and accurate reinstatement has been linked to robust recollection (Kuhl et al., 2011). However, a number of questions remain about the nature of encoding-retrieval reactivation. It is unknown whether encoding-retrieval reactivation reflects processes supporting both encoding and retrieval, and their relation to the reinstatement of memory contents. This study used event-related functional magnetic resonance imaging (fMRI) to investigate the relationship between successful encoding and retrieval, asking whether the recollection of visual context also re-engages regions which supported encoding of the same context.

Different events are processed differently, and are also encoded differently. According to the levels of processing framework, the memory trace is a byproduct of an event’s initial processing (Craik and Lockhart, 1972). Findings consistent with this principle have been reported by numerous fMRI studies using the “subsequent memory procedure,” which compares activity during ongoing tasks according to whether information is later successfully remembered or forgotten. These have shown that the regions associated with later successful item recognition vary as a function of the encoding task or strategy (Otten and Rugg, 2001) and the nature or modality of the encoded material (Otten et al., 2002; Mitchell et al., 2004; Awipi and Davachi, 2008; Park et al., 2008; Prince et al., 2009; Gottlieb et al., 2010). To establish whether the contextual information which is central to an episodic memory is also encoded selectively, encoding activity associated with subsequent contextual retrieval must be compared with that associated with subsequent item recognition when the context is forgotten. Two studies have reported selective SM effects for color and location context which were enhanced by attention to that context (Uncapher et al., 2006; Uncapher and Rugg, 2009). Kuhl et al. (2012) also found that multi-voxel codes distinguishing face from scene context in temporal and frontal cortex predicted subsequent memory for these contextual features. Together, the findings suggest that selective perceptual processing of different features may contribute to the binding of those features as context in episodic memory traces (Meiser and Sattler, 2007).

The contextual features for which encoding selectivity has been demonstrated are similar to those reported to show selective reactivation at the time of retrieval. Despite this convergence, no study so far has assessed whether recollection re-engages regions selectively involved in encoding, as well as processing, distinct types of events. This would suggest that the cognitive operations which support an event’s original encoding are re-engaged during its successful retrieval. Recapitulation is defined as the re-engagement of encoding operations, while reactivation may reflect re-engagement of any processing which took place during the events, whether or not that processing was involved in their encoding. Both encoding and retrieval factors could contribute to recapitulation, and Danker and Anderson (2010) identified two possibilities: one encoding- and one retrieval-based^1^. If cognitive operations which contribute to encoding are integrated as internal context within the memory trace, activity in regions associated with successful encoding will be recapitulated during contextual retrieval when this context is recollected alongside external features of the events. Several studies have reported selective reactivation of online activity when events differed only in the orienting tasks or strategies applied at encoding (Johnson and Rugg, 2007). This implies the recollection of a memory trace comprising internal context as well as the shared external features (Craik and Tulving, 1975). For example, a studied word might be recollected together with a semantically related word if it was encoded using a semantic orienting task, or with a rhyming word if it was encoded using a rhyme generation task. Processes which actually support encoding may form part of this remembered internal context.

Another reason to predict selective recapitulation of encoding operations is that these operations may support retrieval. Episodic retrieval depends critically on cues, and according to the transfer-appropriate processing (TAP) principle is thought to occur when there is sufficient overlap, or match, between the processing of the cue and the cognitive operations used to encode the memory representation (Tulving and Thomson, 1973; Morris et al., 1977; but see Nairne, 2002; Goh and Lu, 2012). Therefore, re-engagement of strategies which helped to encode specific contextual features should increase the probability of recollection of these features because it increases overlap with the corresponding memory traces (Danker and Anderson, 2010).

This study investigated the recapitulation of context-selective encoding operations at retrieval, asking whether regions engaged selectively in the successful encoding of context are also re-engaged at the time of retrieval, and whether this recapitulation of encoding operations is also context-selective. It had two related aims. The first was to extend the handful of earlier findings suggesting that context-selective episodic encoding activity is associated specifically with successful contextual retrieval, as opposed to successful item recognition (Uncapher et al., 2006; Uncapher and Rugg, 2009). The context discrimination task permitted the measurement of context-selective effects at encoding and at retrieval by pairing face and scene context with words in a study phase. In the test phase, studied and unstudied words were retrieval cues. A “guess” response option at test was included for items known to be studied but whose context was uncertain. This helped to ensure sufficient “context forgotten” trials to assess subsequent context memory effects in the encoding phase, as well as reducing the dilution of retrieval success effects by “lucky guesses.” Critically, the design enabled specific contrasts to be used to isolate selective context encoding and retrieval effects for faces and scenes, whilst controlling for encoding and retrieval of the words: in both cases, activity associated with context and item memory (word recognition and context retrieval) was compared with that for item memory alone (word recognition but context forgetting). The prediction was that distinct regions would be engaged during the successful encoding of face and scene context. The study’s second aim was to investigate overlap between context-selective encoding and context-selective retrieval, with the prediction that contextual retrieval would selectively recapitulate encoding operations; i.e., that successful encoding effects and successful retrieval effects would overlap for face and for scene context.

## MATERIALS AND METHODS

### SUBJECTS

Twenty-six volunteers aged 18–30 years (eight women) took part in the study and gave written consent. Eighteen were included in the study (five were excluded prior to fMRI analysis due to poor performance, i.e., <10% correct discrimination of face from scene context; two were excluded due to insufficient forgotten items, and a further one was excluded due to poor data quality; see fMRI Analysis). All were right handed, and reported good health, with no previous significant neurological or systemic illness. The study was approved by the South East Scotland Research Ethics Committee (ref. 11/AL/0323).

### MATERIALS

Stimuli were faces, scenes, phase-scrambled faces and scenes, and words. Faces and scenes were selected from two pools of black and white photographs of 120 faces and 120 scenes (maximum 250 × 250 pixels). Faces were of mixed age and gender including hair, and scenes were of mixed indoor and outdoor locations. Faces were provided by Taylor and the FERET database (Phillips et al., 1998, 2000). Scenes were provided by Taylor and the psychological image collection at the University of Stirling^2^. Following pilot testing in the scanner, 20% phase-scrambled noise was added to the face stimuli to offset their subjectively higher quality and reduce memory performance to obtain sufficient forgotten trials. Words were selected from a pool of 384 selected from a larger set of adjectives with 3–8 letters and Kucera–Francis written frequency of 5–100 per million (Kucera and Francis, 1967)^3^. Each subject’s stimulus lists were selected randomly from the item pools with random allocation of words to face, scene, and unstudied conditions. Scrambled control images for each block were generated from a random subset of that block’s faces and scenes using MATLAB code (v7.6^4^ adapted from Koveski^5^). Each of the six study lists comprised a random ordering of 20 faces and 20 scenes paired with words, and 10 scrambled faces and scrambled scenes, each paired with a non-word comprising five random consonants. Each of the six test lists comprised a random ordering of the 20 words studied with faces in the preceding study list, the 20 words studied with scenes, and 20 unstudied words. An additional 18 faces, 18 scenes, their scrambled counterparts, and 36 words provided practice lists and fillers (two at the start of each study and test phase).

### PROCEDURE

#### Behavioral task

**FIGURE 1:**
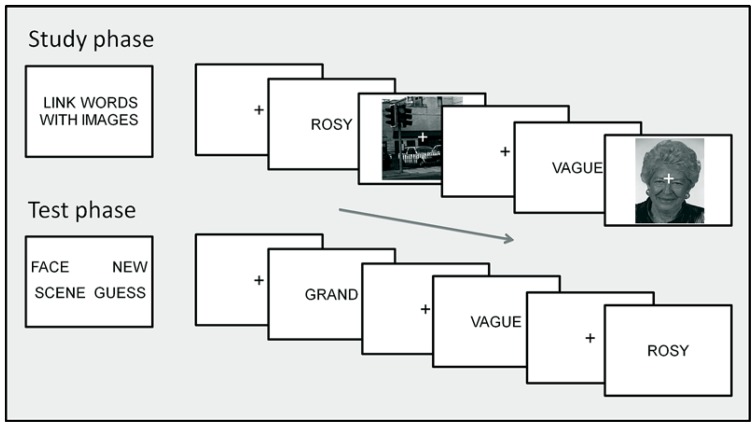
**Task procedure.** The top section illustrates the study phase instructions (left) and two example trials (right) presenting words with scene and face context. The lower section illustrates the test phase instructions (left) and three example trials presenting words previously studied with face and scene context, with scene context and a new word. Text and images on screen are not to scale; see Procedure: Behavioral Task for details of display and timing.

The task is illustrated in **Figure 1**. Subjects completed six study-test blocks in the magnetic resonance imaging (MRI) scanner separated by a brief arithmetic filler task of duration approx. 30 s, which required a button-press response. A prompt for the task and responses appeared on screen prior to the start of each study and test phase. In both phases, stimuli were presented in central vision against a black background (maximum visual angle 9.5° × 9.5°). Words were presented in black upper case “Arial” font. Keypress responses were made with the thumbs and index fingers of both hands using two response button units. Response hands were counterbalanced across subjects. Short practice blocks of both phases were given before entering the scanner.

In the study phases, subjects judged the link between each word and the accompanying face or scene image, rating their goodness of fit on a 4 point scale from “Good fit” to “Poor fit.” No responses were required to the scrambled image control trials. On each trial, the word appeared for 500 ms, followed by a 150 ms blank screen. The face, scene or scrambled image was then presented for 2000 ms with a superimposed central fixation “+,” followed by fixation for 750 ms, giving a total stimulus onset asynchrony (SOA) of 3400 ms.

In the test phases, subjects indicate using button presses whether each word had been presented in the preceding study phase with a face (“Face word”), or with a scene (“Scene word”), whether it had been presented but they could only guess whether with a face or a scene (“Guess”), or whether it was new (“New word”). On each test trial, the word was presented for 1500 ms, followed by the fixation character for 1700 ms, giving a total SOA of 3200 ms. Instructions were to respond as quickly as possible without sacrificing accuracy.

#### MRI data acquisition

A 1.5T GE Sigma MRI scanner (GE Medical, Milwaukee, WI, USA) was used to acquire T2^*^-weighted transverse echoplanar (EPI) images (64x64 3x3 mm^2^ pixels, TR/TE = 2.2 sec/40 ms, flip angle 8°), with blood oxygenation level dependent (BOLD) contrast. Each EPI image comprised 30 3.5 mm-thick axial slices taken every 5 mm (1.5 mm gap), with interleaved acquisition in an ascending direction with coverage of the cerebrum. Two sessions of 633 scans were acquired, including four volumes discarded to allow for T1 equilibration. The ratio of SOA to TR meant that the BOLD impulse response was sampled every 200 ms over trials. Following functional scanning, a T1 structural scan (256 x 256 matrix, 1 x 1 x 1.3 mm^3^ voxels) was acquired.

#### Behavioral analysis

Study trials were classified according to test phase performance into eight encoding conditions of interest: (1) words with face context that was later correctly recalled (“face subsequent context hits”), (2) words with face context that was later incorrectly recalled or could not be recalled and therefore attracted a “guess” response (“face subsequent context misses”), (3) words with face context that were later forgotten and classified as “new” (“face subsequent misses”), (4) non-words with scrambled faces (“scrambled faces”), (5) words with scene context that was later correctly recalled (“scene subsequent context hits”), (6) words with scene context that was later incorrectly recalled or could not be recalled and therefore attracted a “guess” response (“scene subsequent context misses”), (7) words with scene context that were later forgotten and classified as “new” (“scene subsequent misses”), (8) non-words with scrambled scenes (“scrambled scenes”).

Test trials were classified into seven retrieval conditions of interest: (1) words studied with face context that was correctly recalled (“face context hits”), (2) words studied with face context that was incorrectly recalled or could not be recalled and therefore attracted a “guess” response (“face context misses”), (3) words studied with face context that were forgotten and classified as “new” (“face misses”), (4) words studied with scene context that was correctly recalled (“scene context hits”), (5) words studied with scene context that was incorrectly recalled or could not be recalled and therefore attracted a “guess” response (“scene context misses”), (6) words studied with scene context that were forgotten and classified as “new” (“scene misses”), (7) correctly identified new words (“correct rejections”).

Study trials with no response, multiple responses, or responses arriving after the next stimulus were marked as invalid, as were test trials with no-responses, multiple responses, or responses arriving after the next stimulus.

#### fMRI analysis

Preprocessing and statistical modeling of the fMRI data were carried out using SPM8^6^ (r4290). Data quality was checked using outlier detection (slices of variance >5 SD; see Morcom et al., 2010). Problem scans were replaced by the average of the adjacent scans, and modeled as confounds in the design matrix with a “1” at the relevant timepoint in a column of zeros (see below). Problem scans comprised 0.2% of the total. The data were then slice-time corrected, and spatially realigned to the first volume of each run using rigid body transformation. Spatial normalization employed the “new segment” protocol in SPM8 (Ashburner and Friston, 2005). Each subject’s structural scan was coregistered with their mean EPI image prior to estimation of normalization parameters from segmentation of the structural. These parameters were used to re-slice the EPI timeseries to 3x3x3 mm voxels in MNI space, and the resulting images smoothed with an 8x8x8 mm full width half maximum (FWHM) Gaussian kernel.

Statistical analysis employed a two-level summary statistic procedure (Holmes and Friston, 1998; Penny and Holmes, 2006). Covariates for the first-level general linear models (GLMs) for each subject were constructed from sequences of delta functions at the event onset times for each condition, with a constant term for each session. Each sequence of onsets was convolved with a canonical hemodynamic response function (HRF) and its temporal derivative (Friston et al., 1998) to form the two covariates for each condition. Parameter estimates for each covariate were estimated from the weighted least squares fit of the model to the data after prewhitening using an AR(1) plus white noise model (Friston et al., 2002). Data for each session were highpass filtered to 1/128 Hz and scaled to a grand mean of 100 across all voxels and scans within a session. Twelve additional covariates were included for each session to capture residual movement-related artifacts (the six translations and rotations determined during spatial realignment and their between-scan differences).

Data were explored at the first-level by computing single subject contrasts of face and scene context processing effects vs. scrambled controls, at a voxel threshold of *P* < 0.001, uncorrected. One subject who did not show reliable online processing effects on this criterion was excluded from further analysis. HRF amplitude images for each contrast were then computed from the first-level parameter estimate images for the two basis functions (Calhoun et al., 2004; Steffener et al., 2010). These allowed inferences about differential response amplitude without bias or loss of sensitivity from variable latency across conditions and subjects. First-level HRF amplitude images formed the data for the second-level analyses, which treated subjects as a random effect. Details of the group-level models and contrasts are given in the Results (Data Analysis Strategy).

To control the family-wise error (FWE) rate at *P* < 0.05, statistical parametric maps (SPMs) were first thresholded at an uncorrected cluster-defining voxel threshold of *P* < 0.005, and a FWE-corrected cluster extent threshold then applied. This cluster threshold was 44 contiguous voxels, determined using the AlphaSim Monte Carlo simulation tool from AFNI (Analysis for Functional NeuroImaging^7^; Cox, 1996). *A priori* region of interest (ROI) analyses employed SPM8’s small-volume FWE correction within spheres of radius 5 mm (for cortex) and 3 mm (for hippocampus) around coordinates of interest. Except where noted, hypothesis tests were of directional hypotheses and therefore employed unidirectional (*T*-) contrasts (see Results: fMRI Analysis Strategy).

Regions showing overlapping activity at encoding and retrieval were assessed using inclusive masking of the relevant SPMs. In all cases, contrasts to be masked were thresholded at the voxel threshold of *P* < 0.005, and the mask contrasts were thresholded at a voxel threshold of *P* < 0.01. This yielded a conjoint voxel significance of *P* < 0.0005 (Fisher, 1950; Lazar et al., 2002; Uncapher and Rugg, 2005). This test follows the same logic as a conjunction analysis with a global null (conjoint) hypothesis (Friston et al., 1999; Nichols et al., 2005). To obtain a final FWE-corrected cluster threshold of *P* < 0.05, a cluster extent threshold of 11 contiguous voxels was determined using AlphaSim (see above) based on this conjoint cluster-defining threshold, and applied to the resulting masked SPMs.

In the encoding and retrieval analyses, effects of one contrast not shared with another were assessed using exclusive masking at an uncorrected voxel threshold of *P* < 0.05 for unidirectional (*T*-) contrasts and *P* < 0.1 for bidirectional (*F*-) contrasts, to discount voxels showing any hint of the exclusively masked effect (note that when exclusive masking is applied, the higher the threshold applied to the mask, the more conservative the resulting inference from the final masked contrast). Selective effects were those unique to one context, i.e., not shared with the other context. To assess whether regions showing context-selective effects also showed differential activity according to context, directional interaction effects were also assessed in these regions at an uncorrected voxel threshold of *P* < 0.01 [see (Uncapher and Rugg, 2005 and Gottlieb et al., 2010) for similar approaches]. Generic effects were those common to both contexts, with no hint of differential effects across contexts.

Anatomical locations and approximate Brodmann labels of the peaks of suprathreshold clusters were established with reference to the Talairach Daemon (Lancaster et al., 1997, 2000) after conversion of MNI to Talairach-equivalent coordinates (Brodmann, 1909; Talairach and Tournoux, 1988; Brett et al., 2001). Locations were checked by inspection in reference to the group mean structural and EPI images and the MNI reference brain (Cocosco et al., 1997).

#### Data analysis strategy

The whole-brain analyses focused on hypotheses concerning context-selective encoding, context-selective retrieval, and their overlap. Context-selective effects and generic effects – which did not vary according to face and scene context – were both assessed. Only subjects with sufficient (≥12) trials in all critical conditions were included [means (ranges) = 65 (41–97) face context hits, 50 (24–72) scene context hits, 30 (14–62) face context misses, and 39 (18–62) scene context misses]. For each participant, four contrasts entered the group-level analyses: (i) successful context encoding effects for faces (face subsequent context hits – face subsequent context misses) and scenes (scene subsequent context hits – scene subsequent context misses), and (ii) successful context retrieval effects for faces (face context hits – face context misses), and scenes (scene context hits – scene context misses).

Group-level analysis of variance (ANOVA) models for encoding and retrieval incorporated two simple contrasts for each participant, one for face context and one for scene context. Hypothesis tests combined the outcomes of group-level contrasts within these models using inclusive and exclusive masking (see Behavioral Analysis for masking procedures). Analyses were conducted in two stages. First, selective and generic effects were computed for (i) successful context encoding and (ii) successful context retrieval. These were then combined for the overlap analyses.

In the first stage, specific contrasts compared contextual encoding and retrieval with item encoding and retrieval alone. Face- (and scene-) selective encoding success effects (i) (context subsequently remembered > forgotten) were assessed by exclusively masking the outcomes of the group-level encoding success contrasts for faces (or scenes) with those for scenes (or faces). Generic encoding effects were assessed by exclusively masking the outcomes of the group-level average encoding success contrast (faces and scenes) with those of the interaction contrast (faces vs. scenes). Face- (and scene-) selective retrieval success effects (ii; context remembered > forgotten) were assessed by exclusively masking the outcomes of the group-level retrieval success contrasts for faces (or scenes) with those for scenes (or faces). Generic retrieval effects were assessed by exclusively masking the outcomes of the group-level average retrieval success contrasts (faces and scenes) with those of the interaction contrast (faces vs. scenes).

In the second stage, recapitulation, defined as the overlap of selective context retrieval effects with context encoding effects, was assessed by inclusively masking the retrieval contrasts (ii) with the encoding (mask) contrasts (i). To assess selectivity of recapitulation, the overlap between selective context retrieval and selective context encoding was tested both for the matching type of context (e.g., face retrieval with face encoding) and for the other type of context (e.g., face retrieval with scene encoding; see Introduction).

In addition to the whole-brain analyses, context encoding and retrieval were also assessed in *a priori* ROIs centered on bilateral coordinates for the fusiform face area and parahippocampal place area. It was possible that face-selective effects would be detected in right fusiform but generic encoding and retrieval effects were expected in left fusiform due to its known role in word form processing (McCandliss et al., 2003). Talairach coordinates for FFA were taken from (Grill-Spector et al., 2004; *x* = -37, *y* = -42, *z* = -16; *x* = 39, *y* = -40, *z* = -16), and for PPA from (Downing et al., 2006; *x* = -28, *y* = -39, *z* = -6, and *x* = 23, *y* = -45, *z* = -5) and converted to MNI-equivalent coordinates (Brett et al., 2001). Generic contextual SM effects were also expected in the hippocampus (e.g., Eichenbaum et al., 2007). Bilateral hippocampal ROIs were also defined centered on the peaks of clusters implicated in associative encoding of pictorial material in Kim’s (2011) meta-analysis (*x* = -20, *y* = -6, *z* = -14, and *x* = 20, *y* = -10, *z* = -14).

## RESULTS

### BEHAVIORAL FINDINGS

#### Study phase

As the study phase judgments were subjective, their accuracy was not assessed. Analyses of response times (RTs) assessed whether these varied according to subsequent memory, and checked the validity of forming a single subsequent context forgotten condition by combining context miss and context guess trials. Median RTs did not differ reliably for these two trial types for either context (*T* < 1). ANOVA with factors of context (face, scene) and SM (subsequent context hit, context miss, miss) revealed a main effect of material [*F*(1,17) = 17.28, *P* < 0.001, but a non-significant main effect of SM, *F*(1,17) = 2.44, *P* > 0.05; and interaction, *F* < 1]. Responses were faster on face than scene trials regardless of subsequent memory [means (SDs) = 1190 (196) ms and 1292 (220) ms].

#### Test phase

**FIGURE 2:**
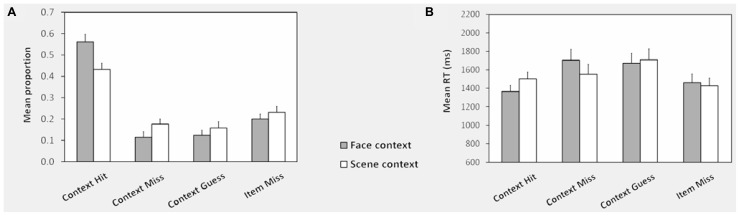
**Context memory performance: test phase. (A)** Shows the mean response proportions in the memory test phase for words studied with face and scene contexts. The proportion of responses in each category is calculated out of the total for each type of context. **(B)** Shows the corresponding mean RTs across subjects for each response category. Note that because there were relatively few face and scene false alarms to new items, RTs were computed collapsed across these conditions. See Behavioral Analysis for definitions of conditions and Test Phase for test phase performance for unstudied items. Error bars represent the standard error of the mean.

Memory test performance for studied items is illustrated in **Figure 2**. Neither accuracy proportions nor median RTs differed reliably for context miss and context guess conditions, so these were collapsed together as context forgotten. ANOVA of accuracy proportions with factors of context (face, scene) and item type (context hit, context miss, miss) revealed a main effect of item type *F*(1.7,28.8) = 22.87, *P* < 0.001, and a context x item type interaction, *F*(1.2,21.5) = 12.57, *P* < 0.001. *Post hoc* pairwise tests (corrected alpha = 0.017) confirmed that context hit proportions were greater for face than scene words, and context miss and miss proportions were greater for scene than face words. The probability of correct context judgment (pC) was corrected for “lucky guesses” to assess the probability of true contextual recollection [pR; see Rugg et al. (1998). Assuming random responding when recollection failed, pR = pC-(1-pR)/3 (the probability of a correct context judgment minus the probability that, in the absence of recollection, such a judgment was the result of a “lucky guess”)]. pR was significantly greater than zero for both faces (mean = .33, *T*(17) = 6.91, *P* < .001) and scenes [mean = 0.15, *T*(17) = 3.41, *P* < 0.05].

Item memory indexed using *Pr* [*P*(source hit, source miss, or source guess) – *P*(new item false alarm)] did not differ according to context (*Pr* = 0.74 for both, *T* < 1; mean proportion of correct rejection of new items = 0.81(SD = 0.13), of face context false alarms = 0.06 (SD = 0.07), of scene context false alarms = 0.03 (SD = 0.04). ANOVA of median test phase RTs revealed a main effect of item type [*F*(1.89,32.1) = 6.24, *P* < 0.005] and a context x item type interaction [*F*(1.23,30.0) = 8.84, *P* < 0.005]. RTs were slower for scene than face context hits [*T*(17) = 5.41, *P* < 0.001], but context misses and misses did not differ.

### fMRI FINDINGS

#### Successful encoding effects

**FIGURE 3:**
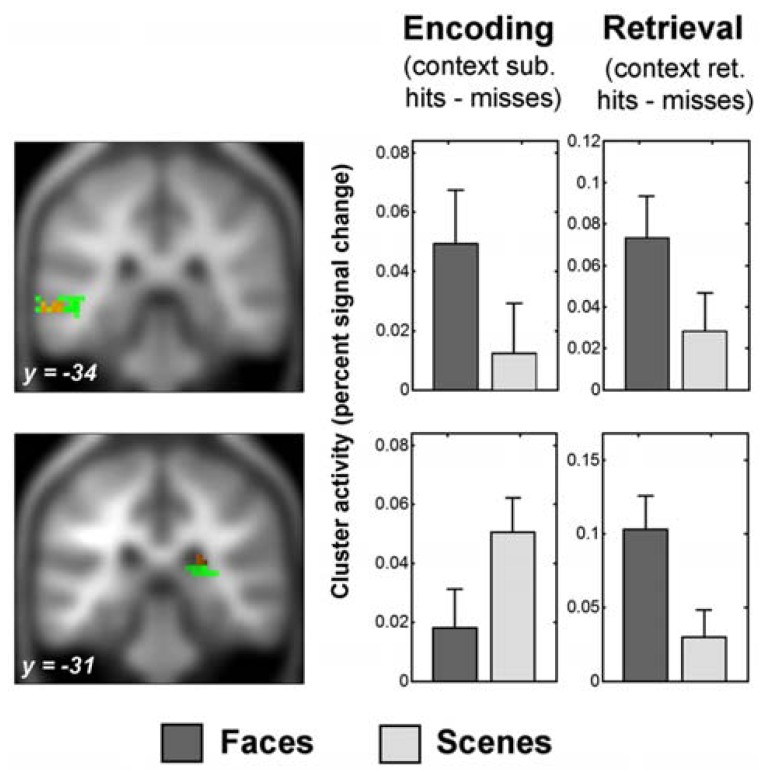
**Recapitulation of encoding activity during face context retrieval.** The sections show the overlap of selective context retrieval effects (overlap shown in red) with selective context encoding effects (shown in green), displayed on the average anatomical T1 image from this sample. Thresholds for display are those used in the overlap analysis (see Behavioral Analysis). Above: left middle temporal gyrus region which showed face-selective encoding activity and face-selective retrieval activity (peak *x* = -63, *y* = -34, *z* = -5). Below: posterior subcortical region which showed scene-selective encoding activity and face-selective retrieval activity (peak *x* = 24, *y* = -31, *z* = 19). Encoding activity and encoding-retrieval activity for this region were masked with a gray matter mask for display purposes (see Successful Encoding Effects and Recapitulation of Encoding Activity at Retrieval for details). Plots show percent signal change successful context encoding effects (left) and successful context retrieval effects (right) averaged across the respective significant clusters of overlapping activity peaks (see Recapitulation of Encoding Activity at Retrieval). Error bars represent standard errors of the mean. Percent signal change was calculated as percent of the cluster overall mean signal change, adjusted for the height of a single trial regressor (http://marsbar.sourceforge.net/faq.html). Y-axes show percent signal change differences for context encoding and retrieval effects as indicated; arbitary units. Note that the scaling is not comparable for the encoding and retrieval plots. See fMRI Analysis and Data Analysis Strategy for details of contrasts, and fMRI Findings for details of clusters.

Face-selective context encoding elicited activity in left inferior frontal gyrus (IFG) and left superior temporal gyrus (see **Table 1** for whole-brain context encoding results, and **Figure 3** for the regions showing recapitulation). Results of the *a priori* ROI analyses are reported only where significant. Inclusive masking with directional interaction contrasts (see fMRI Analysis) indicated that a subset of voxels in left inferior frontal gyrus (LIFG) also showed greater activity for face than scene encoding (4 voxels, peak *x* = -39, *y* = 20, *z* = 4). Scene-selective context encoding elicited activity in the vicinity of the right pulvinar. A subset of this region also showed greater activity for scene than face encoding (18 voxels, peak *x* = 21, *y* = -34, *z* = 16). At the present spatial resolution, this cluster overlaps lateral ventricle and white matter as well as gray matter regions when overlaid on the smoothed averaged T1 image. To clarify its likely origin, gray matter masks were applied using the AAL template in WFU PickAtlas^8^ (Tzourio-Mazoyer et al., 2002; Maldjian et al., 2003) with a dilation factor of 2 voxels (6 mm) to correspond to the applied smoothing kernel of 8 mm FWHM. The cluster included both thalamus (7 voxels) and posterior hippocampus (24 voxels). As predicted, there was also scene-selective encoding activity in the right PPA ROI (4 voxels, peak *Z* = 3.09).

**Table 1:** Encoding success: selective and generic contextual encoding effects.

Location of peak (*x, y, z*)	Peak *Z*	*N* in cluster	Region	Brodmann area
**Face-selective subsequent context memory effects**
-39,	23,	1	3.39	53	Left dorsal anterior insula	BA 13
Includes subpeak:				
-54,	23,	-8	3.14	–	Left inferior frontal gyrus	BA 47
-51,	-49,	-11	3.16	48	Left inferior temporal gyrus	BA 37
Includes subpeak:				
-57,	-40,	1	3.14	–	Left middle temporal gyrus	BA 22
**Scene-selective subsequent context memory effects**
21,	-34,	16	4.74	55	Right posterior hippocampus/pulvinar	–
**Generic subsequent context memory effects**
-30,	5,	37	3.54	125	Left precentral gyrus	BA 6/45
Includes subpeak:				
-57,	20,	22	3.45	–	Left inferior frontal gyrus	BA 9
-15,	26,	40	3.49	106	Left superior frontal gyrus	BA 8/6
-45,	-46,	-14	3.48	91	Left fusiform gyrus	BA 37
-51,	32,	-14	3.45	122	Left inferior frontal gyrus	BA 47

*Regions tabulated show significant (*P* < 0.005, cluster size >44) subsequent memory effect**s** in the whole-brain analysis, using unidirectional (*T*) tests. For selective effects, the subsequent context hits > subsequent context misses effect (*T*) for each context was exclusively masked with the bidirectional (*F*) subsequent context hits vs. subsequent context misses effect for the other context. For generic effects, the average subsequent context hits > subsequent context misses effect (*T*) across both contexts was exclusively masked with the interaction contrast (*F*) comparing subsequent context hits vs. subsequent context misses for face and scene context (see fMRI Analysis for masking procedures, and Data Analysis Strategy). *Z* statistics are given for the final (masked) contrasts. *N* refers to the number of voxels in each cluster, and *x, y* and *z* refer to distances in mm from the origin in MNI space (see fMRI Analysis).*

In addition, generic context encoding effects were observed in regions including left posterior and anterior IFG, left fusiform gyrus (including in the left FFA ROI 6 voxels, peak *Z* = 3.24), the right PPA ROI (5 voxels, peak *Z* = 3.00), the left hippocampal ROI (2 voxels, peak *Z* = 2.89), and medial frontal gyrus.

#### Recapitulation of encoding activity at retrieval

**FIGURE 4:**
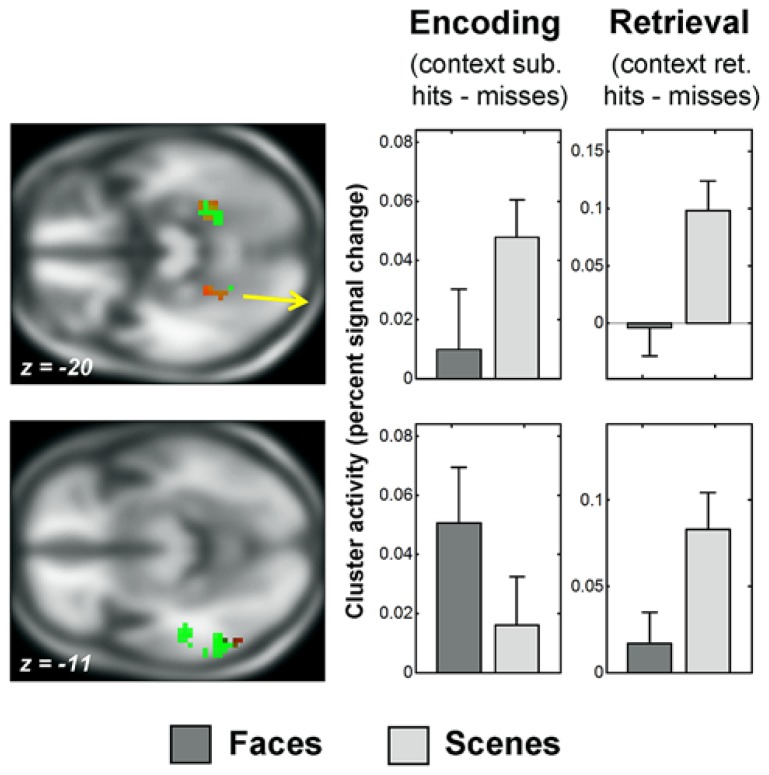
**Recapitulation of encoding activity during scene context retrieval.** The sections show the overlap of selective context retrieval effects (overlap shown in red) with selective context encoding effects (shown in green), displayed on the average anatomical T1 image from this sample. Thresholds for display are those used in the overlap analysis (see Behavioral Analysis). Above: posterior parahippocampal gyrus regions which showed scene-selective encoding activity and scene-selective retrieval activity (peak *x* = -24, *y* = -40, *z* = -20; and *x* = 30, *y* = -46, *z* = -17). Below: left middle occipital/inferior temporal gyrus region which showed face-selective encoding activity and scene-selective retrieval activity (peak *x* = -54, *y* = -61, *z* = -11). See **Figure 2** for information about the parameter estimate plots, fMRI Analysis and Data Analysis Strategy for details of contrasts and fMRI Findings for details of clusters.

Substantial context-selective retrieval activity was present and is summarized in **Table 2** for the whole-brain analysis. The subsets of regions showing recapitulation of successful context encoding activity during successful context retrieval are illustrated in **Figures 3** and **4**. Inclusive masking with tests of directional interaction effects also showed greater activity for face than scene retrieval within all the face-selective regions except for those in left middle and superior temporal gyri, and greater activity for scene than face retrieval within all the scene-selective regions (see **Table 2**; details of interaction overlap available upon request).

**Table 2:** Retrieval success: selective and generic contextual retrieval effects.

Location of peak (*x, y, z*)			Peak *Z*	*N* in cluster	Region	Brodmann area
**Face-selective context retrieval effects**			
21,	-10,	25	4.92	1094	Caudate body	–
-6,	59,	37	4.43	159	Superior frontal gyrus	9
3,	-58,	25	4.23	90	Cingulate gyrus	31
15,	-25,	-17	3.95	45	Midbrain	–
-9,	29,	4	3.88	71	Anterior cingulate	24
-66,	-40,	-5	3.87	46	Middle temporal gyrus	21
60,	-4,	37	3.78	70	Precentral gyrus	6
42,	-22,	-14	3.62	61	Superior temporal gyrus	22
24,	-13,	61	3.51	96	Precentral gyrus	6
-48,	-61,	25	3.57	46	Superior temporal gyrus	39
3,	-82,	-23	3.44	97	Right cerebellum	–
15,	-97,	-8	3.43	65	Cuneus	17
**Scene-selective context retrieval effects**			
-30,	-85,	31	4.79	624	Cuneus	19
12,	-49,	10	4.43	274	Posterior cingulate	29
-9,	-31,	37	4.20	91	Cingulate Gyrus	31
-30,	-40,	-20	4.19	317	Fusiform gyrus	37
21,	29,	-5	3.48	67	Inferior frontal gyrus	47
-6,	-4,	28	3.34	57	Cingulate gyrus	24
**Non-selective context retrieval effects**			
3,	-61,	16	5.69	3789	Posterior cingulate	23
3,	59,	-14	4.74	454	Medial frontal gyrus	11
0,	23,	7	4.68	445	Caudate head	–
-12,	44,	49	4.24	207	Superior frontal gyrus	8
21,	-16,	34	3.9	149	Cingulate gyrus	24
51,	-46,	-20	3.79	46	Fusiform gyrus	37
-33,	-31,	25	3.76	76	Insula	13
-24,	23,	19	3.67	87	Claustrum	–
-30,	35,	-11	3.64	56	Middle frontal gyrus	11
54,	-1,	46	3.6	165	Precentral gyrus	6
24,	-28,	-17	3.33	104	Parahippocampal gyrus	35

*Regions tabulated show significant (*P* < 0.005, cluster size > 44) context retrieval effects in the whole-brain analysis, using unidirectional (*T*) tests. For selective effects, the context hits > context misses effect (*T*) for each context was exclusively masked with the bidirectional (*F*) context hits vs. context misses effect for the other context. For generic effects, the average context hits > context misses effect (*T*) across both contexts was exclusively masked with the interaction contrast (*F*) comparing context hits vs. context misses for face and scene context (see fMRI Analysis for masking procedures, and Data Analysis Strategy). *Z* statistics are given for the final (masked) contrasts. N refers to the number of voxels in each cluster, and x, y and z refer to distances in mm from the origin in MNI space (see fMRI Analysis).*

Face-selective context retrieval activity overlapped (**Figure 3**) with face-selective context encoding activity in left middle temporal gyrus (LMTG, BA21; *x* = -63, *y* = -34, *z* = -5; 12 voxels). It also overlapped with *scene*-selective context encoding activity in superior frontal gyrus (BA8; *x* = -6, *y* = 59, *z* = 40; 11 voxels) and in the vicinity of the right pulvinar (*x* = 24, *y* = -31, *z* = 19; 18 voxels). Inclusive masking with directional interaction contrasts (see fMRI Analysis) showed that the latter region also overlapped (4 voxels) with that showing greater activity for scene than face encoding (see Recapitulation of Encoding Activity at Retrieval). Gray matter masking (see Successful Encoding Effects) also confirmed overlap with a subset of the thalamus (3 voxels) and hippocampal (1 voxel) areas identified in the scene-selective encoding analysis.

Scene-selective context retrieval activity overlapped (**Figure 4**) with scene-selective context encoding activity in bilateral anterior fusiform/posterior parahippocampal gyrus (BA37; *x* = -24, *y* = -40, *z* = -20; 15 voxels and *x* = 30, *y* = -46, *z* = -17; 14 voxels). Inclusive masking with directional interaction contrasts also revealed greater activity for scenes than faces in these regions in a subset of voxels (11 and 7). It also overlapped with *face-*selective context encoding activity in a cluster in left middle occipital and inferior temporal gyri (BA37; *x* = -54, *y* = -61, *z* = -11; 28 voxels). A subset of these voxels (6) also showed greater activity for faces than scenes at encoding.

#### Brain-behavior analysis for other-context recapitulation

*Post hoc* correlation analyses were conducted to assess whether other-context recapitulation (indexed by fMRI context retrieval effects from the pulvinar and left occipitotemporal regions; see Successful Encoding Effects and Recapitulation of Encoding Activity at Retrieval for peak coordinates) was associated with better individual context memory performance (indexed by the average proportion of correct context retrieval for the two contexts). These were both positive, and reliable in pulvinar (Spearman’s ρ = 0.53, *P* = 0.024; for the occipitotemporal peak, ρ = 0.22, n.s.; Bonferroni-corrected α = 0.025).

## DISCUSSION

This study investigated whether episodic retrieval involves recapitulation of processes which were involved in encoding the same events. It assessed regions selectively engaged during successful encoding of face and scene context and the recapitulation of this activity during contextual retrieval, yielding two main findings. First, encoding face and scene context with visually presented words engaged distinct, context-selective regions as well as common regions. This extends previous reports that encoding color and spatial contexts with the same items engages different regions (Uncapher et al., 2006; Uncapher and Rugg, 2009), and is consistent with studies which have shown dissociable neural correlates for the successful encoding of items in different forms and cognitive contexts (Otten and Rugg, 2001; Otten et al., 2002; Mitchell et al., 2004; Awipi and Davachi, 2008; Park et al., 2008; Prince et al., 2009; Gottlieb et al., 2010). Second, a subset of context-selective encoding regions showed reactivation when the same context was later recollected. Critically, this recapitulation did not show the same context selectivity as had been observed at encoding. Instead, the successful retrieval of face and scene context re-engaged regions which had been associated, selectively, with the successful encoding of the other, as well as the same, type of context.

### BEHAVIORAL FINDINGS

The word-image fit judgments took slightly longer for scene than for face context. However, as this difference did not vary according to whether the context was successfully encoded, generic factors, such as the difficulty of processing each kind of context, are unlikely to account for the selectivity of contextual encoding effects. In the test phase, contextual discrimination was above chance, although better for faces than for scenes. Some debrief reports, and fMRI findings discussed below, suggest that a possible reason for this difference was that subjects were better able to find verbal descriptors for the faces than the scenes and consequently to encode them semantically.

An important potential implication of the difference in retrieval difficulty is that subjects might have relied exclusively on recollection of face context, employing “recall-to-reject” to correctly identify words studied with scene context; i.e., judging words as having been studied with scenes if no face context could be recollected, rather than recollecting the scenes. Consistent with this possibility, subjects took ~140 ms longer to correctly identify words studied with scene than face context. However, use of a recollect-faces strategy is inconsistent with the fact that RTs were also ~190 ms slower for face context misses than scene context hits [*T*(17) = 2.87, *P* < 0.05]. If there had been exclusive use of this strategy, responses in the latter two conditions should both have been based solely on attribution of a failure to recollect faces to having studied items with scenes (incorrectly or correctly). It also does not account for the equivalent RTs for context misses for the two conditions, since scene word context misses (incorrectly judged to have been studied with faces) should have been faster (see Test Phase). Retrieval strategy is discussed further below in light of the fMRI findings.

### fMRI FINDINGS

#### Selective encoding

Generic SM effects, equivalent for faces and scenes, were observed in left IFG, hippocampus, premotor cortex, MFG and fusiform gyrus. These regions are known to be involved in successful episodic encoding [for meta-analysis see (Kim, 2011)]. This generic contextual encoding activity presumably reflected processing involved in the binding of the words to visual context independently of the nature of the context. Similar regions have previously been implicated in item encoding and (in the case of left IFG) specifically with verbal encoding, supporting the suggestion that contextual encoding depends in part on efficient item encoding (Gottlieb et al., 2010; Kim, 2011).

Face-selective SM effects were also present, notably in LIFG (between the more anterior and posterior generic encoding regions), as well as LMTG. Across studies, SM effects in LIFG are more likely for verbal than pictorial material (Kim, 2011). In the current study, verbal descriptors may have been more readily accessible for faces than scenes (see above), or words may have been more easily associated with the faces, thus selectively supporting item-context binding when context involved faces. This is consistent with the absence of reliable face context encoding effects in any of the visual cortical ROIs (although this is a null finding). The generic effects in left FFA were expected because faces (and scenes) were encoded with visual words (McCandliss et al., 2003; see Data Analysis Strategy). This is superficially at odds with findings of Kuhl et al. (2012) which implicated both FFA and PPA in face and scene context encoding. However, the discrepancy may be more apparent than real. MVPA analysis may detect responses not apparent at the single voxel level (Norman et al., 2006). The classifier in that study was also trained to distinguish face from scene trials, not predict memory for each separately, so the association of FFA activity with SM may have reflected the encoding of the words with both faces and scenes. As noted above, effective encoding of items may also support their binding to context. Consistent with the current findings, Kuhl et al. (2012) also reported frontal engagement in visual contextual encoding.

The present context-selective SM effects for scenes in right PPA are also consistent with previous findings. A substantial literature links PPA to scene-selective visual processing (Epstein and Kanwisher, 1998; Cichy et al., 2013) as well as scene-selective item encoding (Brewer et al., 1998; Turk-Browne et al., 2006; Hayes et al., 2007; Awipi and Davachi, 2008; Prince et al., 2009; Preston et al., 2010) and two prior studies to scene-selective contextual encoding (Uncapher et al., 2006; Uncapher and Rugg, 2009). These findings support the view that enhanced sensory processing of context can increase the probability of its later recollection (Meiser and Sattler, 2007; Kuhl et al., 2012). However, in the present study additional, generic, contextual SM effects were also observed in right PPA. These may reflect its proposed wider role in coding peripheral visual information, given presentation of both types of context behind centrally presented words (Park et al., 2011). Alternatively, as discussed further below, temporally contiguous scene and face context may have been jointly as well as separately encoded. There was also scene-selective encoding activity in the vicinity of the left pulvinar (posterior thalamus) and posterior hippocampus, which did not include PHG nor overlap with the scene-selective encoding activity in PPA. The pulvinar has not previously specifically been associated with episodic encoding. Scene-selective hippocampal activity may have reflected differential engagement of location-related processes (Nadel et al., 2013). However, given the limited present spatial resolution it is not possible to determine the precise location of this activity.

Although enhanced visual processing of visual context may help its encoding, the distribution of face-selective SM effects also suggests that other kinds of processing can contribute (see also Kuhl et al., 2012). A contribution of a range of distinct cognitive operations to the encoding of different contextual features is also consistent with previous findings of task-, modality- and feature-selective SM effects for items remembered with their context or with high confidence (Otten and Rugg, 2001; Otten et al., 2002; Mitchell et al., 2004; Park et al., 2008; Prince et al., 2009; Gottlieb et al., 2010). The appropriate processing at the time of an event is also likely to depend, in part, on the nature of the later memory test (see Otten, 2007).

#### Recapitulation of encoding activity

As predicted, retrieval of face and scene context was associated with re-engagement of some of the regions associated with its successful encoding. However, although substantial selective retrieval activity was present for both types of context, this involved recapitulation of other-context as well as same-context encoding-related activity. Face context retrieval selectively reactivated the left MTG region which had shown face-selective contextual SM effects, but also re-engaged two regions which had been selectively involved in encoding scene context, in superior frontal gyrus and right pulvinar. Similarly, scene-selective context retrieval re-engaged both posterior PHG, which had shown scene-selective contextual SM effects, and a left occipitotemporal region which had shown face-selective context encoding activity. Importantly, the highly specific context-selective contrasts compared successful context-with-item encoding and retrieval with item-only encoding and retrieval. Both these contrasts – and therefore their overlap – controlled for activity associated with the processing of the words paired with the two contexts (see Kuhl et al., 2012, for a related approach). Furthermore, overlap between context-selective encoding and retrieval effects was tested, rather than overlap between generic context encoding and retrieval effects. Therefore, the other-context recapitulation cannot be explained simply by the re-engagement at retrieval of processes involved in the binding of the words to both visual contexts (see Selective Encoding).

The encoding-based account outlined in the Introduction assumed that encoding operations will be recapitulated when these are recollected as part of the internal context of an event. Both behavioral studies and studies of encoding-retrieval reactivation suggest that the way that events are processed determines how they are encoded, and people recollect this internal context alongside the external features of events (Craik and Tulving, 1975). In the current study, it is easy to see that recollection of encoding operations which had supported encoding of face and scene context may have contributed to the re-engagement of activity in regions associated with successfully encoding the same context, as predicted by Danker and Anderson (2010). If this is correct, why might other-context recapitulation also be observed? One possibility could be that subjects used a recall-to-reject strategy consistently on both face and scene trials. Two observations suggest that this is unlikely to have been the case. First, as discussed above (Behavioral findings), the behavioral findings are consistent, if anything, with the identification of words studied with scenes by the easier recall-to-reject of other words studied with faces. Given the greater difficulty of scene context retrieval, recollection of operations selectively involved in face encoding seems unlikely to have occurred consistently on scene context trials. Second, although the recapitulation of SM effects at retrieval was not context-selective, the contextual retrieval effects themselves overall showed substantial context-selectivity, which would not be expected if both contexts were recollected on both face and scene trials (see Recapitulation of Encoding Activity at Retrieval and **Table 2**).

An intriguing variation on the encoding account is that other-context recapitulation effects may reflect temporal contiguity effects between the two contexts studied on adjacent trials^9^. Work on free recall shows that items studied together are more likely to be retrieved together because shared temporal context binds temporally adjacent items and cues retrieval of each if the other is remembered (Sederberg et al., 2008). It is possible that subjects not only retrieved the context which had been studied with each set of word cues, and the cognitive operations involved in encoding it, but also some of the other-context which had accompanied words studied just before or after them. There were not enough trials in the present study to test this possibility directly by assessing whether other-context recapitulation varied with whether items were encoded adjacent to other context or not. However, two pieces of evidence argue against this account of the data. First, even if triggered by same-context retrieval, retrieval of temporally adjacent other-context would tend to impede context memory performance, reducing the likelihood of observing positive retrieval success effects in these regions (see Kuhl et al., 2011), for evidence of competitive reactivation of face and scene context associated with the same cues, although recapitulation of encoding activity was not assessed). The positive correlation in the present study between other-context recapitulation for items studied with faces and individual context memory performance is also more consistent with the possibility that the other-context recapitulation supports retrieval.

The retrieval-based account described in the Introduction can accommodate the recapitulation of both same- and other-context encoding operations, if these encoding operations are re-engaged in service of retrieval. This assumes that either type of encoding operation could have helped trigger successful recollection of both studied contexts. Since the retrieval task and cues (words) were the same, subjects presumably engaged in attempts to retrieve either kind of context on both types of trials. Correctly identifying a word as having been studied with a face (or scene) may therefore have been supported by a failed attempt to recollect that it had been studied with a scene (or face), as well as by search for and recollection of the studied context. On this account, recapitulation of successful encoding activity at the time of retrieval is consistent with established principles of memory reviewed in the Introduction which assume that recollection is a joint function of encoding and retrieval. Memory representations reflect and incorporate initial processing, and retrieval is successful when this initial processing is recapitulated (Tulving and Thomson, 1973; Morris et al., 1977; Roediger et al., 2002; Rugg et al., 2008). These principles predict that some of the same processes support the encoding and retrieval of particular information, consistent with the pattern observed here. Further studies are needed to directly adjudicate been the encoding and retrieval accounts of the present findings, which are not mutually exclusive, and establish the degree to which recapitulation is transfer-appropriate. Blocking trials by context at encoding (see above) should not impact retrieval-based other-context recapitulation, but manipulation of retrieval cues or instructions, or blocking at retrieval, should do. When instructions target stored information selectively, cue processing re-engages online processing engaged during the targeted events (Johnson and Rugg, 2007; McDuff et al., 2009). By investigating overlap with successful encoding activity, future studies can address whether cues also elicit recapitulation of encoding operations, and whether this is associated with successful recollection.

## CONCLUSION

This study investigated context-selective activity associated with the successful encoding of visual context, and the selective recapitulation of this activity during later contextual retrieval. Distinct selective encoding effects were observed for faces and scene context, extending previous findings for color and location. The distribution of these effects supports the view that enhanced perceptual processing of visual context can selectively support its encoding, but that other factors also contribute. Recapitulation of context-selective encoding activity was observed during contextual retrieval, but with re-engagement of processes previously engaged in other-context as well as same-context encoding. For face context, other-context recapitulation was positively associated with context retrieval success over individuals as well as over trials. These novel findings place constraints on both encoding and retrieval-based accounts of recapitulation. They are most consistent with the view that some of the same processes support contextual encoding and later recollection, and that these processes contribute to encoding-retrieval reactivation.

## Conflict of Interest Statement

The author declares that the research was conducted in the absence of any commercial or financial relationships that could be construed as a potential conflict of interest.

## Footnotes

1 The present study used a relatively short delay encoding-retrieval design, as did many of the reactivation studies reviewed by Danker and Anderson (2010). It is therefore assumed that the relationship between encoding and retrieval activity was not significantly mediated by consolidation processes, although such processes will be likely to have significant impact on reactivation and recapitulation over time scales of hours and longer (see Wang and Morris, 2010).

2 http://pics.psych.stir.ac.uk/

3 http://www.psych.rl.ac.uk/MRC_Psych_Db.html

4 http://www.mathworks.co.uk/products/matlab/

5 http://www.csse.uwa.edu.au/~pk/Research/MatlabFns/

6 http://www.fil.ion.ucl.ac.uk/spm/software/spm8/

7 http://afni.nimh.nih.gov/

8 http://fmri.wfubmc.edu/

9 Thanks to a reviewer for suggesting this possibility.

## Abbreviations

fMRI: functional magnetic resonance imaging
FWHM: full width half maximum
RT: response time
TAP: transfer-appropriate processing
TE: echo time
TR: repetition time
LIFG: left inferior frontal gyrus
SM: subsequent memory.
